# Supplementary material for: The Myeloid Biomarker MS4A6A Drives an Immunosuppressive Microenvironment in Glioblastoma via Activation of the PGE2 Signaling Axis
Source: Int J Mol Sci. 2025 Dec 20;27(1):58. doi: 10.3390/ijms27010058 (PMC12785608; doi:10.3390/ijms27010058)
Supplement: Supplementary file 1 [file ijms-27-00058-s001.zip › ijms-4010774-supplementary.pdf]

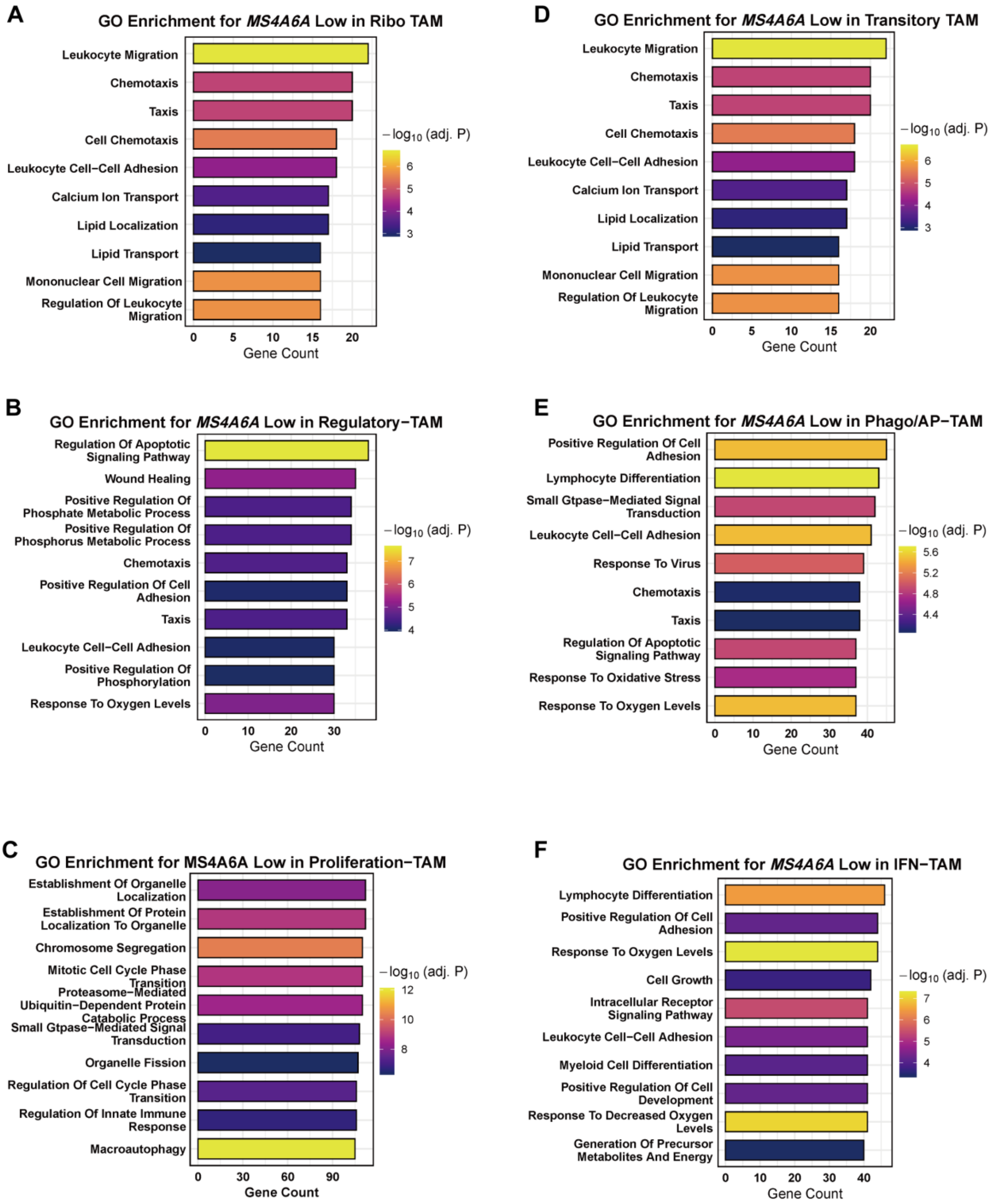

Supplementary Figure S1. Gene Ontology enrichment analysis identifies biological processes associated with low MS4A6A expression across TAM subtypes.

(A) In Ribo-TAM, low *MS4A6A* expression is associated with enhanced leukocyte migration, chemotaxis, and lipid transport, indicating preserved motility and metabolic adaptability.
(B) In Regulatory-TAM, enriched pathways include apoptotic signaling regulation, wound healing, and chemotaxis, reflecting a reparative and immunomodulatory phenotype that may be suppressed

upon *MS4A6A* upregulation.

(C) In Proliferation-TAM, *MS4A6A*-low cells show significant enrichment in organelle localization, chromosome segregation, mitotic cell-cycle transition, and proteasome-mediated protein catabolism, highlighting active proliferation, protein turnover, and metabolic vigor.

(D) In Transitory-TAM, *MS4A6A*-low macrophages are enriched for leukocyte migration, lipid localization, and calcium ion transport, suggesting a dynamic migratory and signaling-intermediate state.

(E) In Phago/AP-TAM, the low-expression group shows enrichment for cell adhesion, lymphocyte differentiation, and viral/oxidative stress responses, consistent with enhanced immune-stimulatory and phagocytic capacities.

(F) In IFN-TAM, enriched pathways include lymphocyte differentiation, intracellular receptor signaling, and myeloid cell development, indicating that interferon-driven immune differentiation programs are attenuated in *MS4A6A*-high conditions.
